# Validation of general job satisfaction in the Korean Labor and Income Panel Study

**DOI:** 10.1186/s40557-017-0167-y

**Published:** 2017-04-05

**Authors:** Shin Goo Park, Sang Hee Hwang

**Affiliations:** 1 Department of Occupational and Environmental Medicine, Inha University Hospital, 27, Inhang-Ro, Jung-Gu, Incheon, 22332 South Korea; 2 Department of Dentistry, Keimyung University School of Medicine, 56, Dalseong-ro, Jung-gu, Daegu, 41931 South Korea

**Keywords:** Job satisfaction, Reliability, Validity

## Abstract

**Background:**

The purpose of this study is to assess the validity and reliability of general job satisfaction (JS) in the Korean Labor and Income Panel Study (KLIPS).

**Methods:**

We used the data from the 17th wave (2014) of the nationwide KLIPS, which selected a representative panel sample of Korean households and individuals aged 15 or older residing in urban areas. We included in this study 7679 employed subjects (4529 males and 3150 females). The general JS instrument consisted of five items rated on a scale from 1 (strongly disagree) to 5 (strongly agree). The general JS reliability was assessed using the corrected item-total correlation and Cronbach’s alpha coefficient. The validity of general JS was assessed using confirmatory factor analysis (CFA) and Pearson’s correlation.

**Results:**

The corrected item-total correlations ranged from 0.736 to 0.837. Therefore, no items were removed. Cronbach’s alpha for general JS was 0.925, indicating excellent internal consistency. The CFA of the general JS model showed a good fit. Pearson’s correlation coefficients for convergent validity showed moderate or strong correlations.

**Conclusion:**

The results obtained in our study confirm the validity and reliability of general JS.

## Background

Job satisfaction is one of the most important factors in the general quality of life, because it is connected closely with working life [1]. There are many definitions of Job Satisfaction (JS), so there are many assessment tools for it. These tools have usually been divided into two types of scale: specific and general scales. General scales are used to estimate the respondent’s general overall feelings about the job. These feelings are expected to predict important behavior, such as quitting or being absent [2].

Since 2002, the nationwide Korean Labor and Income Panel Study (KLIPS) has been conducted annually to collect information on general JS.). The questionnaire of general JS was formulated by specialists based on the Job Satisfaction Index [3]. It consisted of five items rated on a scale from 1 (strongly disagree) to 5 (strongly agree). The general JS of KLIPS has usually been analyzed to provide statistical data for labor policies, but it has not been validated. Validity and reliability are the two fundamental elements in evaluation of a measurement instrument [4].

We investigated the validity and reliability of general JS in the Korean Labor and Income Panel Study (KLIPS).

## Methods

The reliability of the general JS scale was assessed using the corrected item-total correlation and Cronbach’s alpha coefficient. The validity was assessed using confirmatory factor analysis (CFA) and Pearson’s correlation coefficients.

### Data

This study used the data from the 17th wave (2014) of the nationwide Korean Labor and Income Panel Study (KLIPS), which selected a representative panel sample of Korean households and individuals aged 15 or older residing in urban areas. The first survey was launched in 1998, and data have been collected yearly since then. The general JS data have been collected yearly since 2002. The survey was conducted using interviews by trained staff. For the purpose of this study, we selected those subjects having jobs (*n* = 7679).

The survey was conducted by the Korean Labor Institution and was approved by the ethical review board of Statistics Korea. A written informed consent form was obtained from each subject.

### Instrument of general JS

The questionnaire of general JS consisted of five items rated on a scale from 1 (strongly disagree) to 5 (strongly agree). It was formulated by specialists based on the Job Satisfaction Index [3]. The framing question is: “What do you think of your current job? Answer by indicating the extent to which you agree or disagree with each item.”

The five items are:

- “I am satisfied with my current job” (item1),
- “I am enthusiastic about my current job” (item2),
- “I enjoy my current job” (item3),
- “I’m feeling rewarded by my current job” (item4),
- “I want to keep my current job unless there is a good reason for changing” (item5).

### Reliability

The reliability of the general JS scale was assessed using the corrected item-total correlation and Cronbach’s alpha coefficient.

We used the corrected item-total correlation to identify the items that were less reliable and to remove them from the general JS scale. Presumably, a low corrected item-total correlation means that a specific item is less associated with the overall scale and would have lower overall reliability [5]. A correlation coefficient of 0.40 or higher was used as a cut-off for identifying the candidate items [6].

Cronbach’s alpha is the most widely used objective measure of reliability. A threshold of 0.7 was considered acceptable, a value >0.8 good, and a value >0.9 to indicate excellent internal consistency [7].

### Validity

Construct validity refers to the extent to which a test measures the construct it is supposed to measure [8]. A commonly used method to investigate construct validity is confirmatory factor analysis (CFA) [9, 10]. CFA is used when researchers have prior knowledge of latent (underlying) variables and seek to confirm factors that they have found [11].

Since our general JS includes only one theoretical construct, CFA was conducted in order to test whether the one factor model construct was confirmed in this sample.

Several fit indices were selected for the CFA model, including the Root mean Square Error of Approximation (RMSEA), the Comparative Fit Index (CFI) and the Tucker-Lewis Index (TLI). Since the overall *χ*2 fit index is greatly influenced by the sample size, tending to over-reject models with a large sample size, this index was not used when drawing conclusions [12]. For CFI and TLI, a threshold value > 0.9 was considered a good fit [13].

For the RMSEA, on the other hand, a value <0.06 was considered as a good fit, a value <0.08 was considered as an acceptable fit and a value >0.1 led to rejection of the model [14].

Convergent validity was used to assess the construct validity. The convergent validity was evaluated with the Pearson’s correlation coefficients for the theoretically correlated construct (one item general JS). The following question was used to assess the one item JS: “Overall, how satisfied are you with your present job?” The answer is assessed using a 5-point Likert scale that ranged from 1 (very dissatisfied) to 5 (very satisfied). Correlations <0.3 were considered negligible, a value between 0.3 and 0.5 as moderate and a value >0.5 as strong [15].

### Statistical analysis

All analyses were performed with SPSS (IBM Corp. Released 2010. IBM SPSS Statistics for Windows, Version 19.0. Armonk, NY: IBM Corp.).

The reliability of the general JS was assessed using the corrected item-total correlation and Cronbach’s alpha coefficient.

We examined validity of the general JS using CFA and Pearson’s correlations. We evaluated the Pearson’s correlations of our scale (five-items) with a one item scale for convergent validity.

We used multiple goodness of fit tests to test the one factor model, including the Root Mean Square Error of Approximation (RMSEA), the Comparative Fit Index (CFI) and the Tucker-Lewis Index (TLI). We used AMOS17 for CFA.

## Results

Subject characteristics are presented in Table 1. The total number of subjects is 7679 (male 59%, female 41%). The age group is the most frequent in the forties (26.1%). The occupation is the most frequent in the professionals (19.2%) (Table 1).

**Table 1:** Characteristics of respondents

Variables	Number	Percent
Total	7679	
Gender		
Male	4529	59.0
Female	3150	41.0
Age		
≤ 29	614	8.0
30–39	1756	22.9
40–49	2003	26.1
50–59	1825	23.8
≥ 60	1481	19.3
Occupation		
Managers	104	1.4
Professionals	1466	19.2
Clerical Support Workers	1105	14.5
Services Workers	805	10.5
Sales Workers	888	11.6
Skilled Agricultural, Forestry and Fishery Workers	589	7.7
Craft and Related Trades Workers	831	10.9
Plant and Machine Operators and Assemblers	913	12.0
Elementary Occupations	939	12.3

The corrected item-total correlations ranged from 0.736 to 0.837. Therefore, no items were removed. The Cronbach’s alpha for general JS equals 0.925, indicating excellent internal consistency of the measure (Table 2).

**Table 2:** Corrected item-total correlations and Cronbach’s alpha

Items	Corrected item-total correlation	Cronbach’s alpha if Item deleted
Item 1	0.796	0.909
Item 2	0.812	0.906
Item 3	0.837	0.901
Item 4	0.837	0.901
Item 5	0.736	0.921
Cronbach’s alpha	0.925	

The CFA of the general JS model showed good construct validity, with the observed data fitting well with the theoretical model. The CFI, TLI and RMSEA (0.995, 0.990 and 0.063, respectively), indicated a good fit (Table 3).

**Table 3:** Goodness of Fit Indices

*X* ^2^	df	*p*	TLI	CFI	RMSEA
155.614	5	0.000	0.990	0.995	0.063

*df* Degree of freedom, *TLI* Turker-Lewis Index, *CFI* Comparative Fit Index, *RMSEA* Root Mean Error of Approximation

The Pearson’s correlation of the five-item scale with the one-item scale of JS ranged from 0.477 to 0.607, indicating moderate or strong correlations of the measure (Table 4).

**Table 4:** Pearson’s correlation five- item scale with one-item scale of general job satisfaction

Item	Mean ± SD	*r*	*P*
Item 1	3.32 ± 0.755	0.607	0.000
Item 2	3.43 ± 0.755	0.518	0.000
Item 3	3.41 ± 0.739	0.567	0.000
Item 4	3.38 ± 0.757	0.549	0.000
Item 5	3.64 ± 0.765	0.477	0.000

## Discussion

This study is the first to investigate the reliability and validity of the general JS in KLIPS. There are many definitions of general JS. Locke suggested that general JS is the pleasant sentiment derived from the perception (cognition) that the professional activity performed allows one’s personal needs and values linked to the job (behavior) to be satisfied and one’s goals to be achieved [16]. According to Spector, the general JS is the way people “feel” about their job and the aspects characterizing it [17].

The general scales of JS ask the respondent to combine his or her reactions to various aspects of the job into a single integrated response. They assume that some sort of processing takes place and ask for its end product. During this process, the respondent may incorporate other aspects not measured in the facet scales or items [2].

There are various instruments to assess general JS: the job satisfaction index (18 items) of Brayfield and Rothe (1951), Minnesota Satisfaction questionnaire (20 items) of Weiss, Dawis, Engl, and Lofquist (1967), the overall measure (five-items) of Hackman and Oldham (1975), the Facet-free Job Satisfaction (five-items) of Quinn and Staines (1979), the Michigan Organizational Assessment Questionnaire (MOAQ) (3 items) of Cammann, Fichman, Jenkins, and Klesh (1979), Job in General (JIG) (18 items) of Ironson et al. [4, 17].

Although they can be classified differently according to the researchers, criterion validity was usually used when a gold standard is available. Content validity and construct validity were usually used when a gold standard is lacking, as in the case of our study [18].

Content validity is a qualitative type of validity, where the domain of the concept is made clear and the analyst judges whether the measures fully represent the domain [19]. Because there is no statistical test to determine whether a measure adequately covers a content area or adequately represents a construct, the content validity usually depends on the judgment of experts in the field [20]. The items of general JS in this study were formulated by experts by considering the Korean situation based on the Job Satisfaction Index [4].

Construct validity forms an essential part of evaluating validity. Our study used CFA and Pearson’s correlation to assess the construct validity. The fit indices of the one factor model showed a good fit in the CFA of our study.

Two types of assessment of general JS have been conducted annually in KLIPS, the five-item scale and one item scale. The Pearson’s correlation of the five-item scale with the one item scale were used to assess the convergent validity. The result showed moderate or strong correlations.

The main procedures for estimating internal consistency among a number of different questions that are supposed to reflect the same concept are the corrected item-total correlation and alpha reliability coefficients [6]. The corrected item-total correlation was used to identify the items that had less reliable signs and to remove them from the general JS scale. The correlations are all >0.4 in our study, so no items were removed.

The most common method of testing internal consistency is the coefficient alpha [21]. The coefficient alpha is useful for estimating the reliability of the item-specific variance in a unidimensional test [22]. The Cronbach’s alpha of 0.925 obtained in our study showed excellent internal consistency. The occupations held by participants in this study are broadly diverse (Table 1). Therefore, this participant pool may be representative of the general population having jobs in Korea.

The description of the qualitative study conducted in the process of making the questionnaires was insufficient, because no validation was conducted when the instrument of general JS was developed for the first time. Although this study provided various validation processes of general JS, other forms of reliability and validity tests could be required to strengthen its applicability in other populations.

## Conclusion

The results observed in our study confirm the validity and reliability of the general JS.

## Abbreviations

CFA: Confirmatory factor analysis
JS: Job Satisfaction
KLIPS: Korean Labor and Income Panel Study
